# Comparative analysis of the molecular subtype landscape in canine and human mammary gland tumors

**DOI:** 10.1007/s10911-022-09523-9

**Published:** 2022-08-06

**Authors:** Helga Bergholtz, Tonje Lien, Frode Lingaas, Therese Sørlie

**Affiliations:** 1 Department of Cancer Genetics, Institute for Cancer Research, Oslo University Hospital, Oslo, Norway; 2 Department of Preclinical Sciences and Pathology, Faculty of Veterinary Medicine, Norwegian University of Life Sciences, Ås, Norway; 3 Institute of Clinical Medicine, Faculty of Medicine, University of Oslo, Oslo, Norway

**Keywords:** Breast cancer, Molecular subtypes, Comparative models, Canine tumor models

## Abstract

Breast cancers in humans belong to one of several intrinsic molecular subtypes each with different tumor biology and different clinical impact. Mammary gland tumors in dogs are proposed as a relevant comparative model for human breast cancer; however, it is still unclear whether the intrinsic molecular subtypes have the same significance in dogs and humans. Using publicly available data, we analyzed gene expression and whole-exome sequencing data from 158 canine mammary gland tumors. We performed molecular subtyping using the PAM50 method followed by subtype-specific comparisons of gene expression characteristics, mutation patterns and copy number profiles between canine tumors and human breast tumors from The Cancer Genome Atlas (TCGA) breast cancer cohort (n = 1097). We found that luminal A canine tumors greatly resemble luminal A human tumors both in gene expression characteristics, mutations and copy number profiles. Also, the basal-like canine and human tumors were relatively similar, with low expression of luminal epithelial markers and high expression of genes involved in cell proliferation. There were, however, distinct differences in immune-related gene expression patterns in basal-like tumors between the two species. Characteristic HER2-enriched and luminal B subtypes were not present in the canine cohort, and we found no tumors with high-level ERBB2 amplifications. Benign and malignant canine tumors displayed similar PAM50 subtype characteristics. Our findings indicate that deeper understanding of the different molecular subtypes in canine mammary gland tumors will further improve the value of canines as comparative models for human breast cancer.

**Supplementary Information:**

The online version contains supplementary material available at 10.1007/s10911-022-09523-9.

## Introduction

Pre-clinical studies of breast cancer often include experimental rodent models; however, there are important biological differences between humans and rodents with regard to mammary gland tumor development and progression, as well as hormone dependency that complicate the transferability of pre-clinical findings into clinical practice for human cancer [1, 2]. There is, therefore, a need for animal models that mimic human breast cancer more precisely. The dog has emerged as a potential candidate and has been demonstrated to be an efficient model for human cancers [3–6], including breast cancer [7–9].

Mammary gland tumors are common in dogs with an incidence rate twice as high as in humans, but with large differences between breeds [10–12]. In contrast to many rodent models, mammary gland tumors in dogs occur spontaneously without intentional genetic or chemical manipulation, and importantly, they occur in an intact immune environment. There are many similarities between canine and human mammary gland cancers, such as age of onset, hormonal influence on tumor development, disease course, clinical outcome parameters (such as tumor size, clinical stage, and lymph node invasion), mode of metastatic spread, and numerous molecular markers and genetic risk factors [7, 8, 13, 14]. Importantly, dogs often live together with humans and the two species are therefore exposed to many of the same environmental factors that can influence cancer development. There are also differences between the two species; while human breast cancer is dominated by epithelial tumors, canine mammary gland tumors (CMGTs) frequently also contain myoepithelial components [15, 16]. Tumor mutational burden (TMB) is generally lower in canine tumors, and found to be comparable to TMB in pediatric tumors [17, 18]. In addition, diagnosis generally happens later in the progression course in dogs than in humans, since it depends on discovering palpable lesions as opposed to detection by screening mammography in humans.

In human breast cancer, tumors belong to one of several intrinsic molecular subtypes that have different prognostic and predictive impacts [19, 20]. *Basal-like* tumors are predominantly triple negative i.e., they lack expression of estrogen receptor (ER), progesterone receptor (PR) and human epidermal growth factor 2 (HER2). Basal-like tumors are usually highly proliferative and are associated with a poor prognosis. *HER2-enriched* tumors often show high expression of HER2 (also known as erb-b2 receptor tyrosine kinase 2 and encoded by the *ERBB2* gene) and are also poor prognosis tumors; however, these may benefit from HER2-targeted therapy. *Luminal A* tumors are usually ER-positive tumors that proliferate slowly and have a relatively good prognosis, while *luminal B* tumors are also ER-positive, but are more proliferative, and have a significantly worse prognosis than luminal A tumors [21].

The intrinsic subtypes can be determined by the PAM50 method, a nearest centroid classifier in which gene expression of 50 genes is correlated to previously defined subtype centroids [21–23]. The PAM50 method, including the 50 genes and the associated centroids were identified from studies of human breast cancer, however, this method captures core biological characteristics of tumors that are relevant in both humans and dogs. There are few studies on the applicability and relevance of PAM50 subtyping in canine mammary gland tumors; however, in a study by Graim et al., PAM50 subtyping was performed on CMGTs from 16 canine patients [9]. They showed considerable resemblance between canine and human tumors of the same subtype at both the transcriptional and mutational levels.

In a comprehensive study by Kim et al. including gene expression analyses of 158 CMGTs, clustering was performed across the PAM50 genes, but without calling the PAM50 subtypes [18, 24]. Their analysis revealed a strong resemblance between CMGTs and human breast tumors including the well-known, clear distinction between basal-like and luminal-like tumors. In the current study, we utilize this unique data resource that includes both exome DNA and whole-transcriptome RNA sequencing data to thoroughly explore the molecular subtype landscape in canine mammary gland tumors. We use the well-established gene expression-based subtyping method PAM50 and assess its applicability as a tool for stratification of tumors when performing canine/human comparative analyses. Using gene expression, mutation and copy number data we performed a comprehensive subtype-specific comparison of CMGT and human breast tumors from The Cancer Genome Atlas (TCGA) and confirmed that the intrinsic subtypes also represent distinct biology in CMGT. We find that luminal A and basal-like are the two main subtypes in CMGT and underline the necessity of stratifying tumors by molecular subtypes when using CMGTs as models for human breast cancer.

## Material and Methods

### Datasets

#### Canine Mammary Gland Tumor Datasets

Data used in these analyses were generated by Kim et al. at Yonsei University College of Medicine, Seoul, Republic of Korea [18, 24]. In their studies, CMGTs were subject to DNA (whole-exome) and RNA sequencing. The RNA sequencing dataset, including clinical information, was retrieved from NCBI’s Gene Expression Omnibus [25] under the accession number GSE119810 [26]. Clinical characteristics are presented in Supplementary File 1. Variant calling results were downloaded as a VCF file [27] and segmented copy number data were kindly provided by Kim [18]. Details on data pre-processing can be found in Kim et al. and include sequence mapping to the CanFam3.1 reference genome using BWA-MEM2 (DNA) or TopHat (RNA), and variant calling (including filtering out germline variants) using GATK4.0. For RNA sequencing data, FPKM (Fragments Per Kilobase of Transcript per Million) was calculated.

Based on PCA analyses, we identified one sample (CMT.774) as an outlier and hence removed this from the dataset. Mutation and copy number analyses were performed on tumors overlapping with the gene expression dataset. For simplicity, the 13 original histological diagnoses were combined into five main histological categories: *Simple carcinoma*, *complex carcinoma*, *mixed tumor*, *benign* and *other histology*. Both original and new categories are shown in Supplementary File ﻿1.

#### Human Breast Cancer Dataset

Data from the TCGA database [28, 29] were downloaded from the UCSC Xena Platform [30]. The TCGA dataset consists of 1097 human breast tumors.

#### Dataset Merging

To enable cross-species comparison and PAM50 subtyping, the CMGT and TCGA RNAseq datasets were merged. Genes present in both datasets were identified (n = 13,071). Next, a Z-score was calculated per gene in each of the two datasets separately before merging. Principal component analysis (PCA) of the merged data showed no distinct separation according to species.

### PAM50 Subtyping

Intrinsic molecular subtyping was performed by applying the PAM50 method to the merged dataset using centroids for the four main intrinsic subtypes: basal-like, HER2-enriched, luminal A and luminal B obtained from Parker et al. [22, 23]. We excluded the normal-like subtype from this study due to uncertainty of its value as a bona-fide subtype [22, 31, 32]. We identified canine orthologs to the PAM50 genes using the HGNC Comparison of Orthology Predictions (HCOP) search tool [33]. We identified ER-positive (ER^+^) and ER-negative (ER^−^) tumors using Estrogen receptor 1 (*ESR1*) gene expression. *ESR1* showed a distinct bimodal distribution in both cohorts (Supplementary Fig. S1), enabling a cut-off to be set across all tumors. Notably, there was high concordance between ER-status determined by ESR1-expression and ER-status determined by IHC in TCGA (kappa = 0.823). It is well known that PAM50 subtyping may be affected by different distribution of ER + tumors in the training dataset used to calculate the original PAM50 centroids (~60% ER^+^) and test datasets (CMGT/TCGA, ~75% ER^+^) [34, 35]. Hence, we performed gene centering separately for ER^+^ and ER^−^ tumors as described in Lien et al. [32]. We then calculated the Pearson correlation between the gene-centered expression values for the 44 genes and the PAM50 centroids separately for each tumor. The subtype corresponding to the highest correlation coefficient was assigned as the tumor’s PAM50 subtype. Subtype assignments for both cohorts are presented in Supplementary File 2. There was high concordance between our subtyping results and the published subtypes for TCGA tumors (kappa = 0.81) [36].

The CMGT RNAseq dataset included expression of 44 of the PAM50 genes (Supplementary File 3). The six genes not present (*BIRC5*, *CXXC5*, *FOXC1*, *KRT17*, *MIA* and *NAT1*) were therefore removed from the analyses. To investigate the impact of removing six genes from the subtype centroids, we performed subtyping of the TCGA RNAseq data separately with 50 and then with 44 genes. These analyses revealed a high correlation between the subtype correlation coefficients obtained from the two gene lists (Pearson correlations, *r* > 0.96 for all subtypes). We therefore concluded that removing these genes would not notably impact the results of the subtyping. We also compared the results when subtyping the TCGA cohort separately and when merged with the CMGT cohort. Here too, the subtype correlation coefficients were strongly correlated across all subtypes (Pearson correlation, *r* > 0.96 for all subtypes).

### Gene Expression Analyses

#### Proliferation Score

The proliferation score was calculated as the mean of the standardized expression of 10 proliferation-associated genes: *CCNB1, CDC20, NUF2, CEP55, NDC80, MKI67, PTTG1, RRM2, TYMS, UBE2C* [37]. The eleventh gene from the original proliferation signature (*BIRC5*) was not present in the canine dataset.

#### Immune Score

Immune genes were obtained from Nanostring’s Canine IO panel [38]. 650 out of the 800 genes overlapped between the immune gene list and the CMGT expression dataset. The immune score was calculated as the mean of the standardized expressions of the 650 immune genes. Genes representing specific immune cell types were selected from the gene annotation supplied by Nanostring.

#### Single Sample Gene Set Enrichment Analyses

Single sample gene set enrichment analyses (ssGSEA) were performed on the merged gene expression dataset (CMGT and TCGA) in GenePattern using the ssGSEA module and the Hallmarks of Cancer (HOC) gene sets from the Molecular Signatures Database [39–41].

#### Independent Validation Data

Two gene expression datasets of canine mammary gland tumors were retrieved from the Gene Expression Omnibus [25] for validation purposes. GSE20718 [42, 43] is gene expression data obtained from the Affymetrix Canine Genome 2.0 array and includes 27 mammary gland tumors; 13 with lymph node metastases and 14 without. 39 of the PAM50 genes were available in this dataset. GSE136197 [9, 44] consists of 63 benign and malign tumors from 16 dogs and was obtained by RNA sequencing. In this dataset, 43 of the PAM50 genes were available for analysis.

### Mutation Analyses

In the CMGT cohort, mutations were annotated using the Ensembl Variant Effect Predictor (VEP) [45] with the CanFam3.1 Genome Assembly [46]. We included in the analyses only non-synonymous mutations of coding regions, and we excluded ten genes characterized as FLAGS (frequently mutated genes) [47]. The tumor mutation burden (TMB) was represented by the number of mutations per tumor. The Cosmic Cancer Gene Census (tier 1 and 2) was downloaded from cancer.sanger.ac.uk [48]. TCGA mutation data was downloaded from the UCSC Xena Platform.

### Copy Number Analyses

Segmentation of CMGT copy number data is described in Kim et al. [18]. The cut-off for amplification was set to segment mean > 0.2 and for deletion < -0.2. A copy number aberration index was calculated separately for each tumor as the proportion of the sequenced genome above or below the cut-off. TCGA copy number data was downloaded from the UCSC Xena Platform.

### Statistical Tests

Statistical analyses were conducted in RStudio version 4.0.5 [49, 50]. Fisher’s Exact Test was used to test associations between the distribution of subtypes in the canine and human cohorts, to test associations between the canine subtypes and histological grade, histopathological diagnosis, neuter status and lymph node invasion, and to test associations between subtype and mutation of specific genes. Kruskal Wallis tests were performed to test for differences in TMB and copy number aberration index across all subtypes, while Mann Whitney U-tests were performed for pairwise comparisons between subtypes. Mann Whitney U tests were also performed to compare ssGSEA signatures between subtypes and between species. All tests were two-sided. For representation in a heatmap, data were clustered using Euclidean distance as the distance metric and complete linkage as the clustering method. Cohen’s kappa coefficient was used for calculating concordance between ER-status based on IHC vs. ESR1 expression in TCGA and for comparing subtype results with subtypes already published for TCGA.

### R Packages

The downloaded RNAseq data were annotated with ENSCAF-IDs and refGenome v1.7.7 [51] was used for gene annotation. VCF files were analyzed using the package vcfR v1.12.0 [52]. Heatmaps were created using Complex Heatmaps v2.7.10 [53] and other plots were created using ggplot2 v3.3.3 [54]. Additional packages used were circlize v0.4.12 [55], factoextra v1.0.7 [56], ggpubr v0.4.0.999 [57], IRanges v2.24.1 [58], vcd v1.4.9 [59] and reshape2 v1.4.4 [60].

## Results

### PAM50 Subtype Distribution and Clinical Properties of Canine Mammary Gland Tumors

**Fig. 1:**
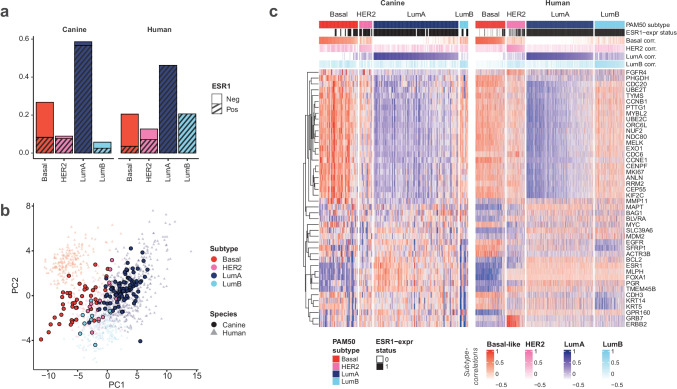
PAM50 subtypes in canine and human mammary gland tumors **a**: Distribution of PAM50 subtypes in CMGTs (n = 157) and the TCGA cohort (n = 1097). Hatched area indicates ER^+^ tumors. **b**: Principal component analyses based on the PAM50 genes. Analysis was performed on the TCGA data with CMGT data overlaid for comparison. First principal component (PC1) on the x-axis; second principal component (PC2) on the y-axis. **c**: Cluster heatmap showing expression of the 44 PAM50 genes present in the CMGT dataset. Tumors are shown in columns (ordered by species, subtype, and subtype correlation) and genes are shown in rows. Clustering of genes was performed using *Euclidean* as distance metric and *complete* as clustering method. Top annotation depicts PAM50 subtype, ER status and correlation coefficients for the four subtype centroids. Data is gene-centered

PAM50 subtyping was performed on the merged RNAseq dataset, which included all canine tumor samples (CMGT, n = 157) and TCGA human breast tumor samples (TCGA, n = 1097). In the canine cohort, all four subtypes were present, and the relative pattern of subtype distribution was comparable to the distribution in the human cohort with minor differences: There was a higher proportion of luminal A and basal-like tumors, and correspondingly fewer luminal B and HER2-enriched tumors in the CMGT compared to the TCGA cohort (Fig. 1a). The proportion of ER^+^ tumors was nearly equal between the species: 75.2% and 76.4% in the canine and human cohorts, respectively. Most luminal A canine tumors were ER^+^, similar to human tumors, while a larger proportion of basal-like canine tumors were ER^+^ compared to basal-like human tumors (Fig. 1a, hatched areas indicate ER^+^).

Next, we investigated the association between CMGTs of different PAM50 subtypes and histopathological diagnosis, histological grade, lymph node invasion and neuter status (Table 1). The histopathology differed significantly between the subtypes (Fisher’s Exact Test, p = 0.002). Most benign tumors were of the Luminal A subtype, as were most complex carcinomas, while most basal-like tumors were simple carcinomas although many simple carcinomas were Luminal A. When including only the tumors considered malignant by histopathology (n = 112), there was still a significant difference between histopathology and subtype (Fisher’s Exact Test, p = 0.004). Among the malignant tumors there was a significant association between the PAM50 subtype and histological grade (Fisher’s Exact Test, *p *< 0.001), with a higher proportion of grade 3 and a lower proportion of grade 1 tumors of the basal-like subtype, while the opposite was true for luminal A tumors. The association between subtype and lymph node invasion was significant (Fisher’s Exact Test, *p* < 0.001) with a higher degree of lymph node invasion in basal-like cancers compared to the other subtypes. We found no association between neuter status and subtype (Fisher’s Exact Test, p = 0.89).

**Table 1:** Clinical characteristics of PAM50 subtypes in CMGT. Number of tumors of each subtype (columns) and clinical characteristics (rows) are shown. Total number of tumors and *P*-values obtained from Fisher’s Exact Test for each clinical characteristic are also indicated. Clinical data obtained from Kim et al. [18, 24]

	**Basal-like**	**HER2-enriched**	**Luminal A**	**Luminal B**	**Total**	***P*-value**
	42 (26.8%)	14 (8.9%)	92 (58.6%)	9 (5.7%)	157	
***Histopathological diagnosis***					157	*p* = 0.002
* Benign*	6	5	31	3	45	
* Mixed tumor*	4	1	8	0	13	
* Complex carcinoma*	6	1	26	0	33	
* Simple carcinoma*	23	7	25	3	58	
* Other histology*	3	0	2	3	8	
***Histological grade (malignant)***					112	*p* < 0.001
* 1*	7	4	48	2	61	
* 2*	9	4	8	2	23	
* 3*	20	1	5	2	28	
***Lymph node invasion***					157	*p* < 0.001
* Absent*	30	13	89	9	141	
* Present*	12	1	3	0	16	
***Neuter status***					157	*p* = 0.89
* Intact*	26	10	63	6	105	
* Neutered*	15	4	28	2	49	
* NA*	1	0	1	1	3	

### Subtype Correlation Coefficients Reveal Relevant Tumor Biology Characteristics

PAM50 subtyping is a nearest-centroid method which, in addition to categorical subtype assignment, yields correlations to all centroids that reveal additional insights into tumor biology [61]. Overall, we observed a similar picture in both cohorts; tumors of the basal-like and luminal A subtypes showed a relatively high correlation to their assigned subtype and a relatively low second highest subtype correlation coefficient (Table 2 and Supplementary Fig. S2). The basal-like and luminal A correlation coefficients were negatively correlated in both species, i.e., tumors with high correlation to the basal-like centroid showed correspondingly low correlation to the luminal A centroid. In HER2-enriched and luminal B subtypes, the picture was less distinct, with low correlation to all subtypes. This was particularly evident in the canine dataset. The subtype assignments of HER2-enriched and luminal B canine tumors are therefore unreliable, due to similar correlation coefficients to several centroids. Importantly, even though most basal-like canine tumors were distinctly basal-like, in general, they showed a lower correlation to the basal-like centroid and were in closer proximity to other centroids compared to the human tumors. The benign tumors in the CMGT cohort did not show lower correlation coefficients to their assigned subtype than the malignant CMGT tumors.

**Table 2:** Subtype correlation coefficients in canine and human tumors for each PAM50 subtype. Interquartile range is shown in parentheses

	**Canine**	**Human**
**Basal-like**	0.45 (0.36–0.57)	0.66 (0.60–0.79)
**HER2-enriched**	0.25 (0.22–0.31)	0.57 (0.43–0.67)
**Luminal A**	0.56 (0.38–0.65)	0.55 (0.45–0.70)
**Luminal B**	0.38 (0.31–0.39)	0.42 (0.33–0.49)

### Comparable Gene Expression Landscape in Canine and Human Mammary Gland Tumors

To further compare the gene expression landscape of canine and human mammary gland tumors of different subtypes, we performed principal component analysis based on the 44 overlapping PAM50 genes using the human dataset. Projecting the canine data onto the human principal components (Fig. 1b) revealed a similar picture in the two species, although with a noticeable shift of the canine basal-like tumors towards the luminal tumors. To explore the contribution of the individual genes to the subtyping, we performed a separate PCA analysis on the canine tumors and compared the results from the two species. The first principal component represented 49% of the variance in both species and there was high compliance between the species with regard to each gene’s contribution to the first principal component (Pearson correlation, r = 0.89, *p* < 0.001) (Supplementary Fig. S3). In both species, the genes with the highest contribution to the first principal component were those involved in cell proliferation, a known distinction between basal-like and luminal human tumors [20].

**Fig. 2:**
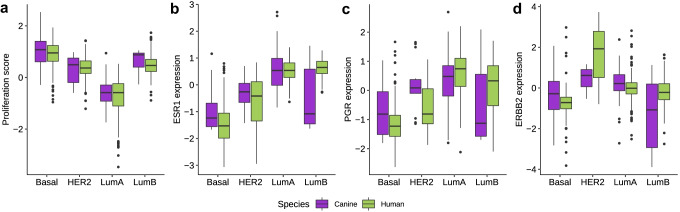
Gene expression characteristics in CMGTs and TCGA tumors by PAM50 subtype **a**: Proliferation score, **b**: Estrogen receptor 1 (*ESR1*) expression, **c**: Progesterone receptor (*PGR*) expression, **d**: erb-b2 receptor tyrosine kinase 2 (*ERBB2*) expression (encoding HER2). Boxplots illustrate the median (middle line) and interquartile range (box); whiskers indicate 1.5 × IQR above and below the box

Cluster analysis of the tumors based on the 44 PAM50 genes revealed similar gene expression patterns in the two cohorts (Fig. 1c). Proliferation-associated genes were distinctly differentially expressed between the subtypes in both species; low expression in luminal A and high expression in basal-like tumors (Fig. 2a). Also, the genes encoding the hormone receptors ER and PR showed a similar profile between the species with generally low expression in basal-like tumors and high expression in luminal A tumors (Fig. 2b, c). In contrast, *ERBB2* and other genes (such as *GRB7*) typically highly expressed in tumors of the HER2-enriched subtype showed species-specific differences, as the canine HER2-enriched tumors lacked distinct overexpression of *ERBB2* and *GRB7* (Figs. 1c and 2d). A separate analysis of the benign CMGT tumors showed that they displayed gene expression subtype characteristics roughly in line with the malignant CMGT tumors (Supplementary Fig. S4).

To validate these findings, we performed a similar cluster analysis across the PAM50 genes in two separate gene expression datasets obtained from canine mammary gland tumors (Supplementary Fig. S5). In both cases, clustering showed distinct separation between the tumors displaying a basal-like phenotype and the tumors of a luminal-like phenotype, similar to our observations in the main dataset (Fig. 1c). Neither of the datasets contained tumors with high expression levels of ERBB2, also in line with the initial results. In one of the datasets (Klopfleisch et al.) [43], about 50% of the tumors were associated with regional lymph node metastases at the time of resection (Supplementary Fig. S5a) and a majority of these showed a basal-like gene expression profile in line with our main results. The second dataset (Graim et al.) [9] consists of multiple tumors from each patient, including a substantial number of benign tumors, and none of those showed a distinct basal-like phenotype (Supplementary Fig. S5b), also in line with our findings in the main dataset.

### Variation in Immune Signatures Between Species

To explore in greater depth the biological processes that characterize CMGT subtypes and how they compare to the corresponding human breast tumor subtypes, we performed ssGSEA on the HOC gene sets in the Molecular Signature Database (Supplementary File 4) [40]. Several gene sets showed very similar profiles in canine and human tumors with distinct differences between the subtypes. For instance, gene sets related to cell cycle regulation (e.g., MYC targets, G2M checkpoint and E2F targets) and WNT/β-catenin signaling were significantly more highly expressed in basal-like tumors compared to luminal A tumors in both species (Supplementary Fig. S6a–d, Mann Whitney U tests: *p* < 0.001 for all basal-like vs. luminal A comparisons). Other gene sets, however, showed diverging expression patterns between canine and human tumors, particularly for the basal-like subtype (Supplementary Fig. S6e–h). Interestingly, a majority of these were related to immune responses, such as the TNFα-signaling via NFΚB, Inflammatory response, and Interferon gamma*-* and Interferon alpha responses*,* which were all markedly more active in human basal-like tumors compared to canine basal-like tumors (Mann Whitney U tests, *p* < 0.001 for all canine vs. human comparisons).

To elaborate on these findings, we investigated differences in gene expression of 650 immune related genes [38]. An immune score derived from these genes was significantly lower in canine basal-like tumors than human basal-like tumors, while no significant differences were seen between canine and human in tumors of the other subtypes (Supplementary Fig. S7a). Hierarchical clustering of basal-like tumors across the immune genes showed that most canine tumors clustered together with human tumors with low immune score (Supplementary Fig. S7b). There was no correlation between the immune score and correlation coefficient to the basal-like centroid in basal-like tumors, thus; lower immune score in canine tumors could not be explained by the overall higher correlation to the basal-like centroid in the human tumors (Supplementary Fig. S7c). Genes specific for T-cells (e.g. CD3D) and macrophages (CD68) were lower expressed in canine compared to human basal-like tumors, while genes highly expressed in mast cells (e.g. CPA3 and MS4A2) were higher expressed in canine basal-like tumors compared to the human counterpart. Moreover, genes expressed in regulatory T cells (FOXP3) and cytotoxic cells (GZMB) were higher expressed in human basal-like tumors, likewise were genes encoding the immune checkpoints proteins PD-1 (PDCD1) and PD-L1 (CD274) (Supplementary Fig. S7d).

Immune scores were also calculated for tumors in the two validation datasets and results were similar to our findings in the main dataset. In the data from Klopfleisch et al., there was a strong positive association between high immune score and lymph node metastasis, and the metastatic tumors were more frequent among those with a basal-like profile (Supplementary Fig. S5a). In the data from Graim et al., samples with high immune score were evenly distributed across the basal-like and luminal-like cluster. (Supplementary Fig. 5b).

### Similar Association Between Subtype and PIK3CA and TP53 Mutations

**Fig. 3:**
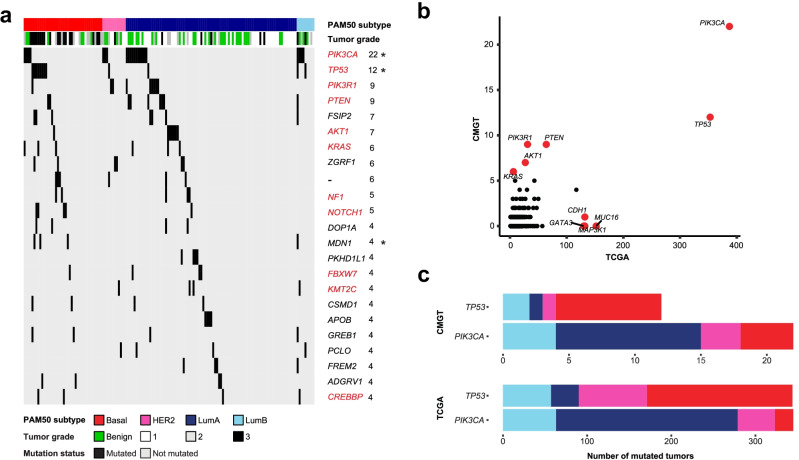
Subtype specific mutation profiles of CMGTs and TCGA tumors **a**: Frequency and subtype association of all genes with four or more mutations across the CMGT dataset. Tumors are ordered by subtype in columns and genes are shown in rows. Top annotation depicts PAM50 subtype and tumor grade. The number of tumors carrying mutations is noted to the right of the gene symbol. Genes present in the Cosmic Cancer Gene Census are shown in red and genes with significantly different mutation frequencies between the CMGT subtypes are marked with an asterisk. One gene was uncharacterized with Ensembl-ID ENSCAFG00000018773 and is marked "-". **b**: Mutation frequency in TCGA (x-axis) and CMGT (y-axis) of genes present in the Cosmic Cancer Gene Census. Genes commonly mutated in either TCGA, CMGT or both are highlighted (red). **c**: Subtype distribution of tumors with *TP53* and *PIK3CA* mutations in CMGT and TCGA

In the canine tumor cohort, we identified 2318 non-synonymous mutations in the coding regions of 1873 genes. Twenty-three genes carried mutations in four or more tumors (Fig. 3a). Of these, 11 genes were previously found to be implicated in cancer and present in the Cosmic Cancer Gene Census [48]. *PIK3CA* and *TP53* were the most frequently and second most frequently mutated genes, respectively, in the canine cohort, which was also the case in the TCGA cohort (Fig. 3b). However, other than these two genes, there were noticeable differences between the species. Some genes with relatively high mutation frequencies in the TCGA cohort (e.g., *GATA3*, *MUC16* and *MAP3K1*) were not mutated in the canine tumors, while genes such as *PIK3R1*, *PTEN*, *AKT1* and *KRAS* were more frequently mutated in canine tumors compared to human (Fig. 3b).

Among the canine tumors, there was a significantly different distribution of tumors with *PIK3CA* and *TP53* mutations between the subtypes (Fisher’s Exact Test, p-values: *PIK3CA* = 0.04, *TP53* < 0.001) with more *PIK3CA* mutations in luminal tumors and more *TP53* mutations in basal-like tumors (Fig. 3c). The same pattern is found in human tumors (Fisher’s Exact Test, *p*-values: *PIK3CA* < 0.001, TP53 < 0.001) [28]. Interestingly, 27% of the benign tumors carried mutations in PIK3CA, while no benign tumors were TP53 mutated (Fig. 3a).

We found that TMB was higher in human breast tumors compared to canine, in line with previous literature [17, 18], and this was true for all subtypes. In human breast cancer, the TMB varies between the PAM50 subtypes [62]. Among the canine tumors, although there was no significant *global* difference in the mutation burden between the subtypes (Kruskal–Wallis Test, p = 0.11), we did identify a significant difference in the TMB between basal-like and luminal A subtypes (Mann Whitney U Test, p = 0.015) (Supplementary Fig. S8a). In the human cohort, the global difference in mutation burden was significant between the subtypes (Kruskal–Wallis Test, *p* < 0.001) and the most pronounced difference was seen between basal-like and luminal A tumors (Mann Whitney U Test, *p* < 0.001) (Supplementary Fig. S8b).

### Comparable Subtype Specific Copy Number Profiles in Canine and Human

**Fig. 4:**
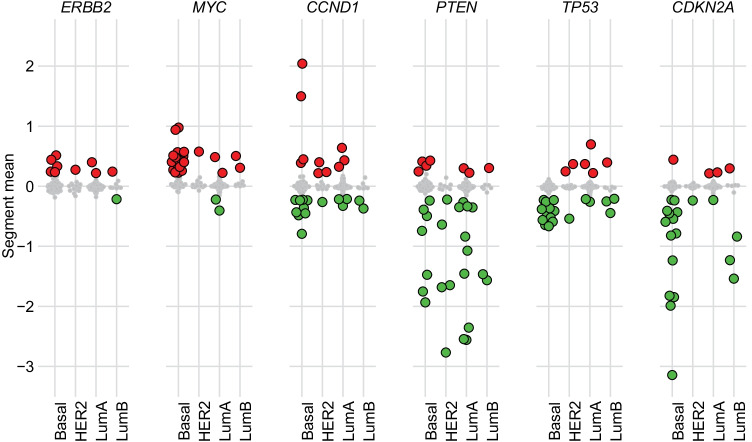
Copy number profiles of cancer-relevant genes in CMGT; Estimated copy number across PAM50 subtypes is illustrated with subtypes on the x-axis and segment mean (representing the copy number) on the y-axis. Tumors with amplifications (segment mean > 0.2) are indicated in red; tumors with deletions (segment mean < -0.2) are indicated in green

We analyzed segmented copy number profiles from the canine tumors and found distinct differences between the subtypes (Supplementary Fig. S9). The aberration index quantifying the proportion of the genome carrying aberrations was significantly different between the subtypes (Kruskal Wallis test, *p* < 0.001) (Supplementary Fig. S10). Basal-like tumors were, in general, highly aberrant with changes across the whole genome, while the luminal A tumors carried distinctly fewer changes. The paucity of aberrations in luminal A tumors was not the result of a large proportion of benign tumors, as these had copy number aberrations comparable to malignant luminal A tumors (data not shown). Similar to human breast cancer [63], the canine basal-like and luminal B subtypes carried markedly more aberrations than the luminal A tumors. However, for the HER2-enriched subtype there were noticeably fewer copy number aberrations compared to that seen in human breast cancer. HER2-enriched tumors are characterized by distinct copy number patterns, often including high-level amplification of the *ERBB2* locus on chromosome 17q1.2 in humans [64]. In dogs, the *ERBB2* gene is located on chromosome 9. There were no tumors with high-level *ERBB2* amplification and no overrepresentation of *ERBB2* amplifications in canine HER2-enriched tumors compared to the other subtypes (Fig. 4).

We also investigated other driver genes that are commonly affected by copy number changes in human breast cancer (Fig. 4). *MYC* amplification, for instance, in humans is most commonly found in tumors of the basal-like subtype [65], and has previously been reported in canine mammary gland tumors with a simple carcinoma histology [66, 67]. In the CMGT-dataset we also found amplifications of *MYC* predominantly in tumors of the basal-like subtype. In addition, *CCND1* is commonly amplified in human luminal tumors [68]. We found *CCND1* amplified in some cases, but there was no subtype-specific pattern. Among the known breast cancer-related tumor suppressor genes, we found *PTEN* deletions in CMGTs of all subtypes, *TP53* deletions in basal-like and luminal B tumors and *TP53* amplifications in a few tumors of the HER2-enriched and luminal A subtypes, while deletions of *CDKN2A* (p16) was mostly seen in basal-like CMGT, similarly to human breast cancer [28].

## Discussion

The dog is presumed to be a valuable comparative model for human breast cancer, but there is a need for increased knowledge about the molecular similarities as well as differences between mammary gland tumors in the two species. The PAM50 subtypes are defined for human breast carcinomas; however, in a comparative setting when exploring dogs as models, it is important to determine the relevance of these subtypes also in canine mammary gland tumors using the same method in both species. Here, we have studied the incidence and characteristics of the PAM50 subtypes in a cohort of canine mammary gland tumors. Overall, the PAM50 subtypes captured molecular subtype characteristics in dogs that are well known from human breast cancer. As in human breast cancer, there was a distinct dichotomy between basal-like and luminal canine tumors that could not solely be explained by hormone receptor status.

Nearly 60% of the CMGTs were of the luminal A subtype. These showed high correlation to the luminal A subtype centroid at a level comparable to human luminal A tumors and relatively low correlation to all other subtype centroids. Canine luminal A tumors were, in general, similar to human luminal A tumors with high expression of luminal epithelial genes such as *ESR1*, *PGR*, *FOXA1* and *MLPH* and low expression of genes involved in cell proliferation. *PIK3CA* mutations were more common in canine luminal A tumors than in other subtypes as is also the case in human tumors. The luminal A tumors carried markedly fewer mutations than basal-like tumors. This, combined with fewer copy number aberrations, indicates a more stable genome in canine luminal A tumors compared to basal-like tumors. The CMGT cohort includes both benign and malignant tumors and most benign tumors were of the luminal A subtype. The benign tumors displayed characteristics in line with their PAM50 subtype, both with regards to proliferation, ESR1 expression, mutations and copy number aberrations. This result is in line with the findings of Sørenmo et al. who proposed benign tumors as precursors of malignant tumors in canines [69].

Tumors of the basal-like subtype constituted 27% of the canine cohort and showed gene expression characteristics well known from analyses of human basal-like tumors. They expressed low levels of luminal epithelial genes and high levels of multiple proliferation-associated genes indicating that these are highly proliferative tumors. The basal-like CMGTs were more often high-grade tumors and of the simple carcinoma histology type compared to luminal A tumors, similar to human breast cancer [70]. However, in contrast to human breast cancer, where basal-like tumors paradoxically are associated with less lymph node invasion than luminal tumors [71], there was a higher number of lymph node-positive cases among canine basal-like tumors compared to the other subtypes. This finding was also evident in one of the independent validation datasets that consisted of approx. 50% locally metastasized tumors. Hence, together with noticeably lower expression of gene signatures related to immune cells in canine basal-like tumors compared to human, this indicates intrinsic differences between canine and human basal-like tumors that might be explained by different immune responses. Exploring species-specific differences in the immune response towards tumors could generate valuable knowledge, especially in view of the emergence of immune therapy in breast cancer treatment [72]. In addition, the basal-like canine tumors had markedly more copy number aberrations than the luminal A tumors in particular, which fits well with what is known about human breast cancer [28]. Canine basal-like tumors also encompassed slightly higher mutation frequencies compared to other subtypes and carried several subtype specific aberrations such as mutations and deletions of *TP53*, amplifications of *MYC* and deletions of *CDKN2A*, all known features of bona-fide basal-like human tumors [28].

In our study, 8.9% of the CMGTs were characterized as HER2-enriched, however, correlation to the HER2-centroid was low for these tumors. *ERBB2*-amplifications in canine tumors were only low level, and were not restricted to tumors of the HER2-enriched subtype. This indicates that the HER2-enriched subtype does not have the same prevalence and relevance in CMGTs, confirming results from several previous studies [18, 66, 73]. Similar to the HER2-enriched subtype, luminal B did not emerge as a definite subtype in the canine cohort. Luminal B tumors were characterized by high expression of genes involved in cell proliferation, low expression of basal keratins and a high copy number aberration count, but displayed large variation in the expression of luminal epithelial genes, *ERBB2,* as well as in TMB.

The canine samples in this study include mammary gland tumors from predominantly small companion dogs. To obtain a more comprehensive and nuanced overview of the potential of dogs as models for human breast cancer, these findings should be validated in an RNA sequencing dataset including tumors from larger dogs and working breeds; however, such datasets of sufficient size do not yet exist. Nevertheless, we were able to validate our main findings in two smaller independent datasets. For this study, we merged the canine and human cohorts and performed subtyping on the merged datasets. Additionally, subtyping was performed using 44 out of the original 50 genes. These factors, however, do not seem to have affected the subtyping results significantly. Different chromosomal composition in dogs and humans complicates comparative copy number analyses. Nevertheless, our study shows that such analyses need to be performed stratified by subtype.

## Conclusions

CMGTs are highly heterogeneous biologically, and represent an unmet potential for modeling human breast cancer. Our study identifies many similarities between mammary gland tumors of dogs and humans, but also discovers important differences and emphasizes that the molecular subtypes should be taken into account when considering dogs as models for breast cancer. Generally, we found a high degree of similarity between canine and human tumors across the four main intrinsic subtypes, but we also pinpointed differences that are important when considering dogs as comparative oncology models. Knowledge obtained from canine/human comparative studies may contribute towards facilitating individualized treatment in dogs suffering from mammary gland tumors and such studies could therefore be of relevance and interest to both the veterinary and human medical communities.

## Supplementary Information

Below is the link to the electronic supplementary material.

**Fig. S1 Distribution density of** ***ESR1*** **expression in canine tumors (purple) and human tumors (green)** *ESR1* expression on the x-axis and kernel density estimate on the y-axis. A common cut-off (vertical line at x = -0.765) was calculated across all tumors in both datasets. This corresponds to the point of minimum density between the *ESR1*-low and *ESR1*-high expression tumors. (PDF 220 KB)

**Fig. S2 Comparison of PAM50 subtypes and subtype centroid correlation coefficients in canine (upper panel) and human (lower panel) tumors** Samples are shown on the x-axis and subtype correlation to each centroid (colored by subtype) is shown on the y-axis. Tumors are ordered by subtype and subtype correlation. (PDF 536 KB)

**Fig. S3 Contribution of individual PAM50 genes to the first principal component in CMGT and TCGA** Principal component analysis was performed based on the PAM50 genes for CMGT and TCGA separately. The contribution of the variables to the first principal component is shown on the x-axis for TCGA and on the y-axis for CMGT. R and P-values are obtained from the Pearson correlation. (PDF 138 KB)

**Fig. S4 Gene expression characteristics of benign and malignant CMGT by PAM50 subtype a:** Proliferation score, **b:** Estrogen receptor 1 (ESR1) expression, **c:** Progesterone receptor (PGR) expression, **d:** erb-b2 receptor tyrosine kinase 2 (ERBB2) expression (encoding HER2). Boxplots illustrate the median (middle line) and interquartile range (box); whiskers indicate 1.5 × IQR above and below the box. (PDF 183 KB)

**Fig. S5 Hierarchical clustering across the PAM50 genes in two independent canine datasets a:** 39 of the PAM50 genes were available in the validation dataset GSE20718 (Klopfleisch et al.) based on Affymetrix gene expression arrays. Tumor samples are shown in columns, genes in rows. Top annotation depicts histological grade, lymph node invasion and immune score **b:** 43 of the PAM50 genes were available in the validation dataset GSE 136197 (Graim et al.) based on RNA sequencing. Tumor samples are shown in columns, genes in rows. Top annotation depicts tumor type and immune score. (PDF 379 KB)

**Fig. S6 Expression of Hallmark gene signatures by PAM50 subtype a–d:** Hallmark gene signatures that differed significantly between basal-like and luminal A subtypes in both species (Mann Whitney U test, p < 0.001). **e–h:** Hallmark gene signatures that differed significantly between canine and human basal-like tumors (Mann Whitney U tests, p < 0.001). Single sample gene set enrichment results for all signatures are presented in Supplementary File 4. Boxplots illustrate the median (middle line) and interquartile range (box); whiskers indicate 1.5 × IQR above and below the box. (PDF 198 KB)

Fig. S7 Immune characterization of CMGT and TCGA a: Immune score by PAM50 subtype in CMGT and TCGA. **b:** Cluster heatmap showing expression of 650 immune genes in basal-like CMGT and TCGA tumors. Tumors are shown in columns and genes are shown in rows. Clustering of genes was performed using Euclidean as distance metric and complete as clustering method. Top annotation depicts species and immune score. **c:** Immune score vs. correlation to basal-like centroid in basal-like CMGT and TCGA tumors. Color indicates species. Correlation coefficient and p-value obtained by Pearson correlation. **d:** Expression of genes characteristic for immune cells in CMGT and TCGA by PAM50 subtype. P-values obtained from Wilcoxon tests comparing expression in canine and human tumors of same subtype are indicated above (* < .05, ** < 0.01, *** < 0.001, **** < 0.0001). Boxplots illustrate the median (middle line) and interquartile range (box); whiskers indicate 1.5 × IQR above and below the box. (PDF 7.48 MB)

**Fig. S8 Tumor mutation burden in a: CMGT and b: TCGA tumors by PAM50 subtype** Subtype is shown on the x-axis and log2 of mutation count (number of coding mutations) is shown on the y-axis. P-values are obtained from Mann Whitney U tests comparing tumors of basal-like and luminal A subtypes. Boxplots illustrate the median (middle line) and interquartile range (box); whiskers indicate 1.5 × IQR above and below the box. (PDF 119 KB)

**Fig. S9 Genome-wide copy number frequencies by PAM50 subtype in CMGT** Genomic position is shown on the x-axis. The y-axis shows the frequency of losses (green) or amplifications (red) in the four subtypes separately. Cut-off for amplification was set at segment mean > 0.2 and for deletion <-0.2. (PDF 1.90 MB)

**Fig. S10 Copy number aberration count by PAM50 subtype in CMGT** Subtype on the x-axis and log2 of the number of aberrations on the y-axis. Boxplots illustrate the median (middle line) and interquartile range (box); whiskers indicate 1.5 × IQR above and below the box. (PDF 196 KB)

Supplementary file11 (XLSX 16 KB)

Supplementary file12 (XLSX 138 KB)

Supplementary file13 (XLSX 15 KB)

Supplementary file14 (XLSX 962 KB)

## Data Availability

Data used in this study are publicly available. The gene expression data can be accessed from the NCBI GEO database under accession number GSE119810 [26]. Clinical information and variant calling data can be downloaded from https://doi.org/10.6084/m9.figshare.c.4543784.v1. Raw data are available from the Sequence Read Archive IDs (SRP159466 and SRP159481) [74, 75].

## Abbreviations

CMGT: Canine Mammary Gland Tumor
ER: Estrogen receptor
ERBB2: Erb-b2 receptor tyrosine kinase 2
ESR1: Estrogen receptor 1
IHC: Immunohistochemistry
HER2: Human Epidermal Growth Factor 2
HOC: Hallmarks of cancer
PCA: Principal Component Analysis
PR: Progesterone receptor
ssGSEA: Single Sample Gene Set Enrichment Analyses
TCGA: The Cancer Genome Atlas
TMB: Tumor Mutation Burden
