# Supplementary figures and images for: Comparative analysis of the molecular subtype landscape in canine and human mammary gland tumors
Source: J Mammary Gland Biol Neoplasia. 2022 Aug 6;27(2):171–83. doi: 10.1007/s10911-022-09523-9 (PMC9433360; doi:10.1007/s10911-022-09523-9)

FIGURE S1

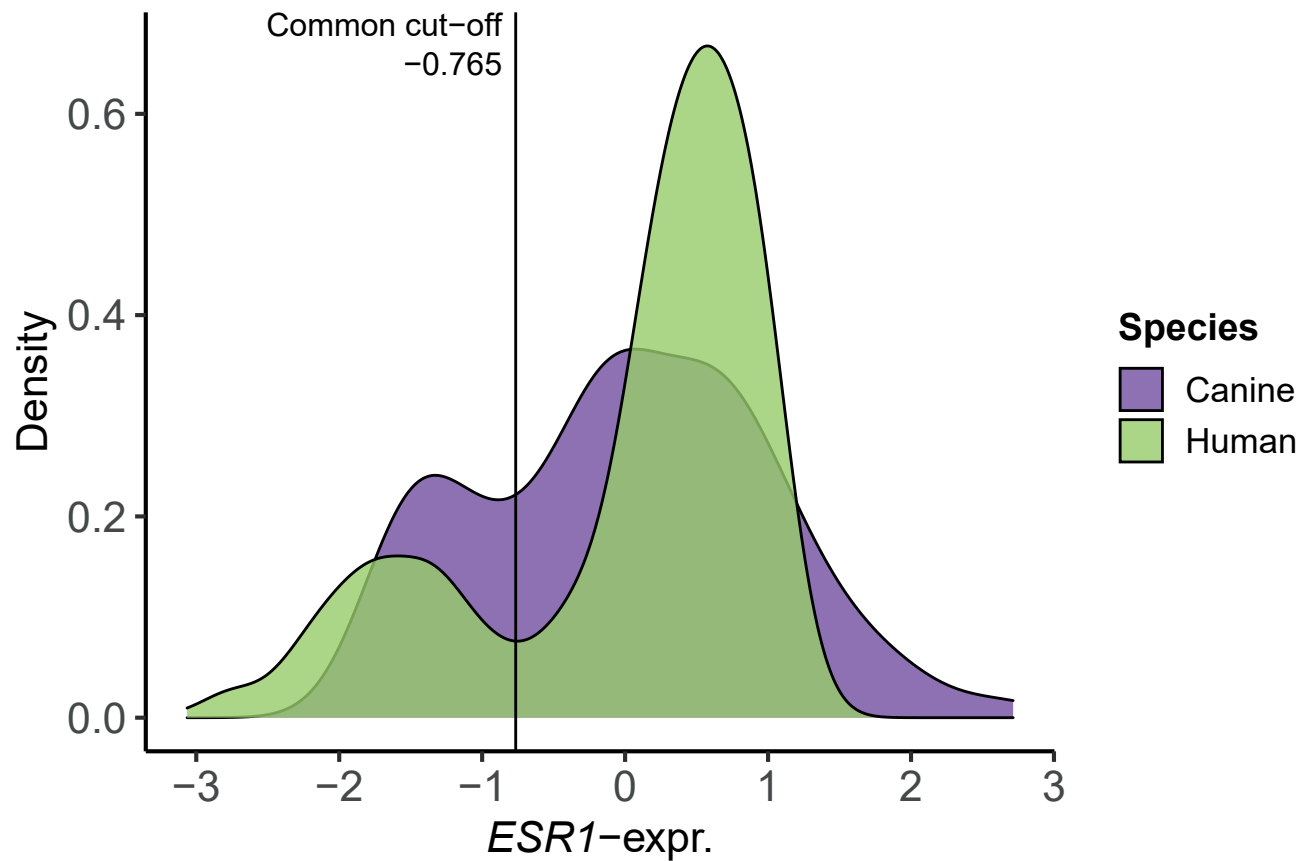

Supplement: Supplementary file 1 — Fig. S1 Distribution density of ESR1 expression in canine tumors (purple) and human tumors (green) ESR1 expression on the x-axis and kernel density estimate on the y-axis. A common cut-off (vertical line at x = -0.765) was calculated across all tumors in both datasets. This corresponds to the point of minimum density between the ESR1-low and ESR1-high expression tumors. (PDF 220 KB) [file 10911_2022_9523_MOESM1_ESM.pdf]

FIGURE S2

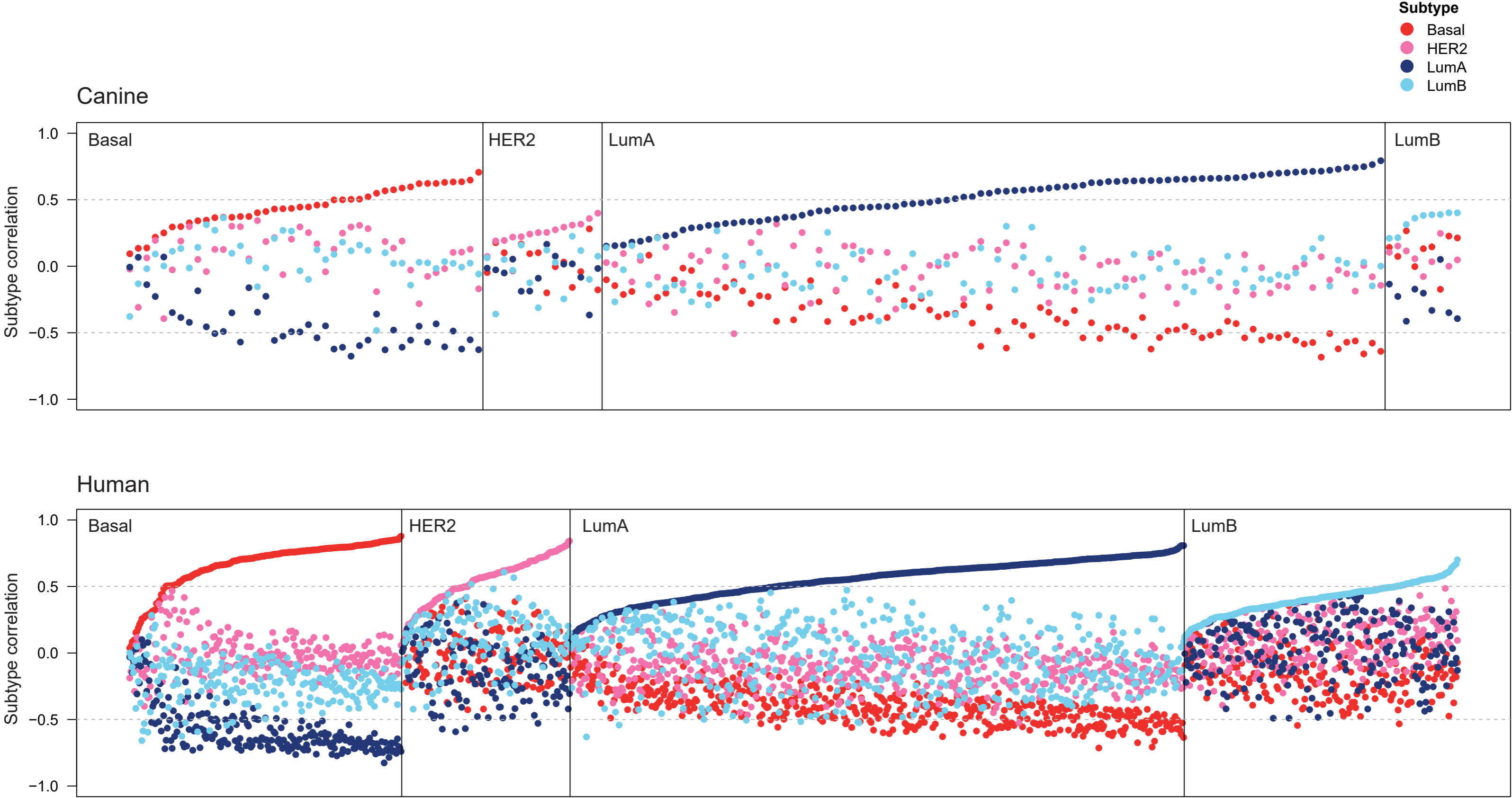

Supplement: Supplementary file 2 — Fig. S2 Comparison of PAM50 subtypes and subtype centroid correlation coefficients in canine (upper panel) and human (lower panel) tumors Samples are shown on the x-axis and subtype correlation to each centroid (colored by subtype) is shown on the y-axis. Tumors are ordered by subtype and subtype correlation. (PDF 536 KB) [file 10911_2022_9523_MOESM2_ESM.pdf]

FIGURE S3

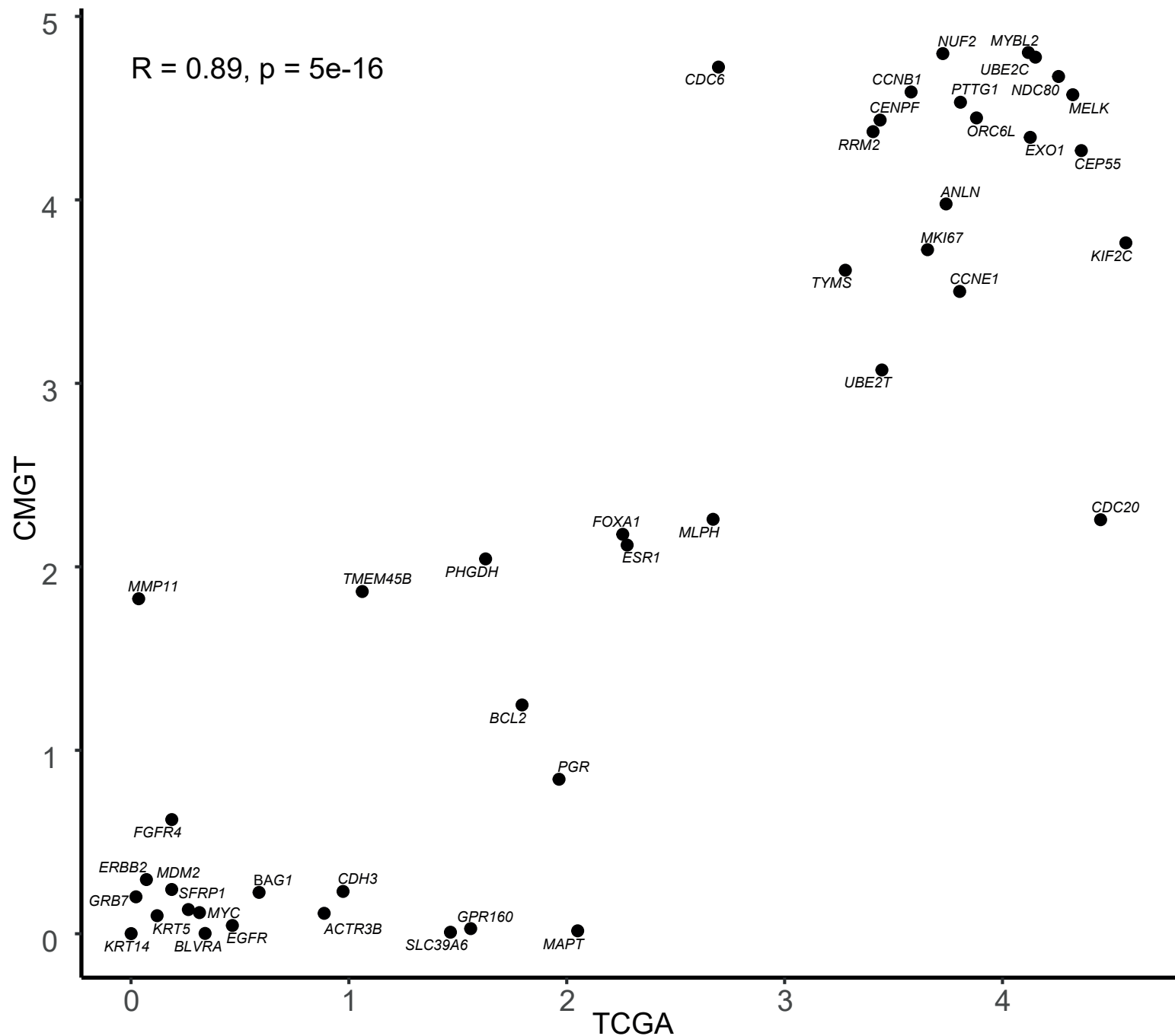

Supplement: Supplementary file 3 — Fig. S3 Contribution of individual PAM50 genes to the first principal component in CMGT and TCGA Principal component analysis was performed based on the PAM50 genes for CMGT and TCGA separately. The contribution of the variables to the first principal component is shown on the x-axis for TCGA and on the y-axis for CMGT. R and P-values are obtained from the Pearson correlation. (PDF 138 KB) [file 10911_2022_9523_MOESM3_ESM.pdf]

FIGURE S4

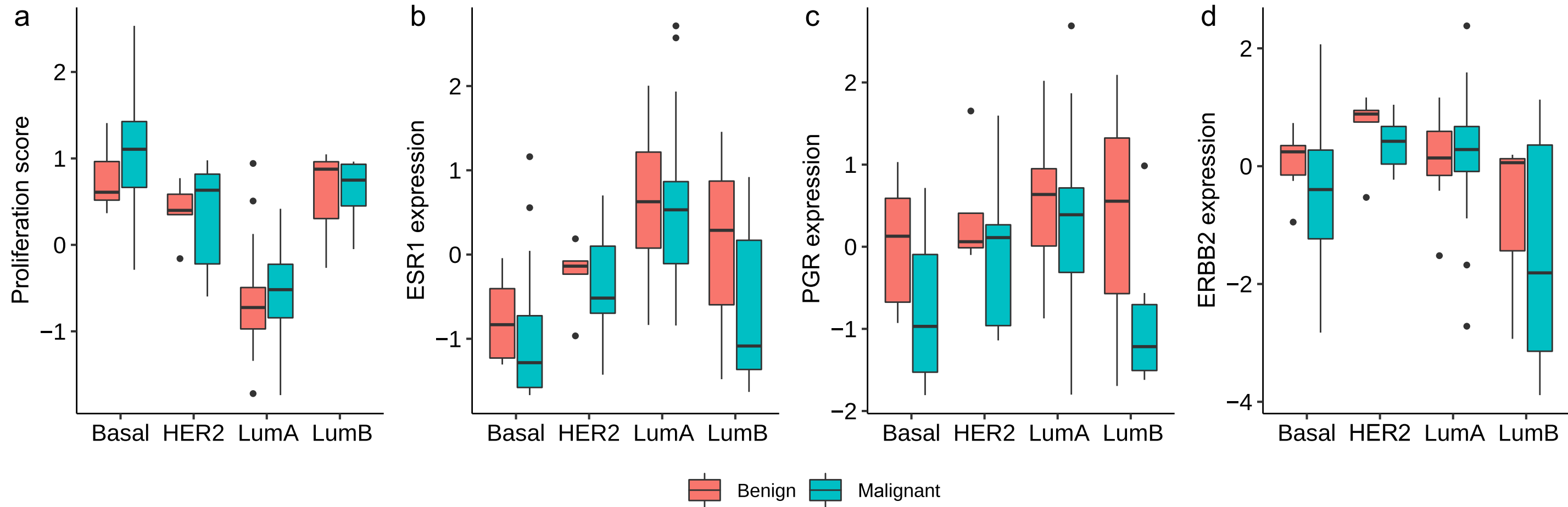

Supplement: Supplementary file 4 — Fig. S4 Gene expression characteristics of benign and malignant CMGT by PAM50 subtype a: Proliferation score, b: Estrogen receptor 1 (ESR1) expression, c: Progesterone receptor (PGR) expression, d: erb-b2 receptor tyrosine kinase 2 (ERBB2) expression (encoding HER2). Boxplots illustrate the median (middle line) and interquartile range (box); whiskers indicate 1.5 × IQR above and below the box. (PDF 183 KB) [file 10911_2022_9523_MOESM4_ESM.pdf]

FIGURE S5

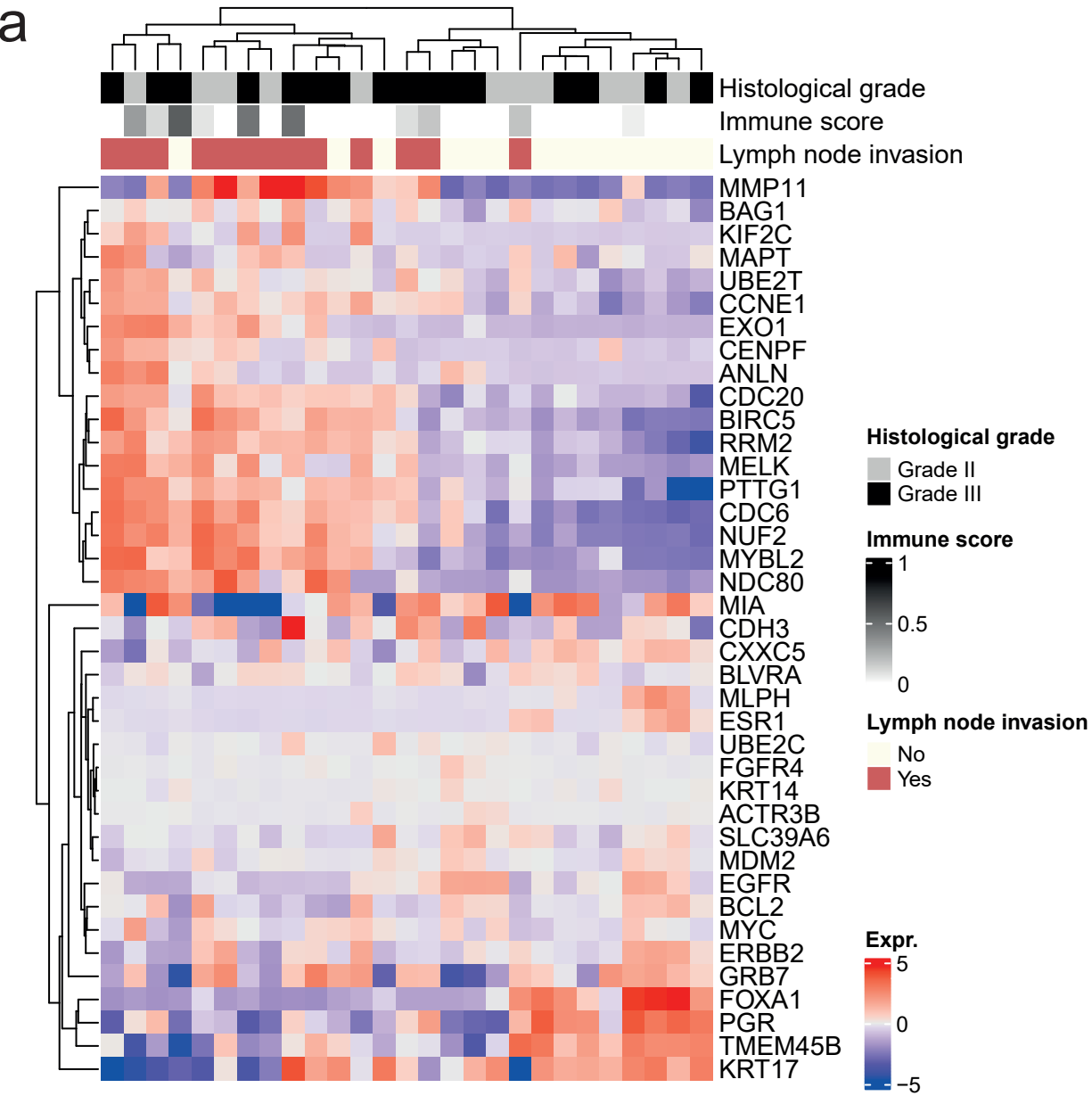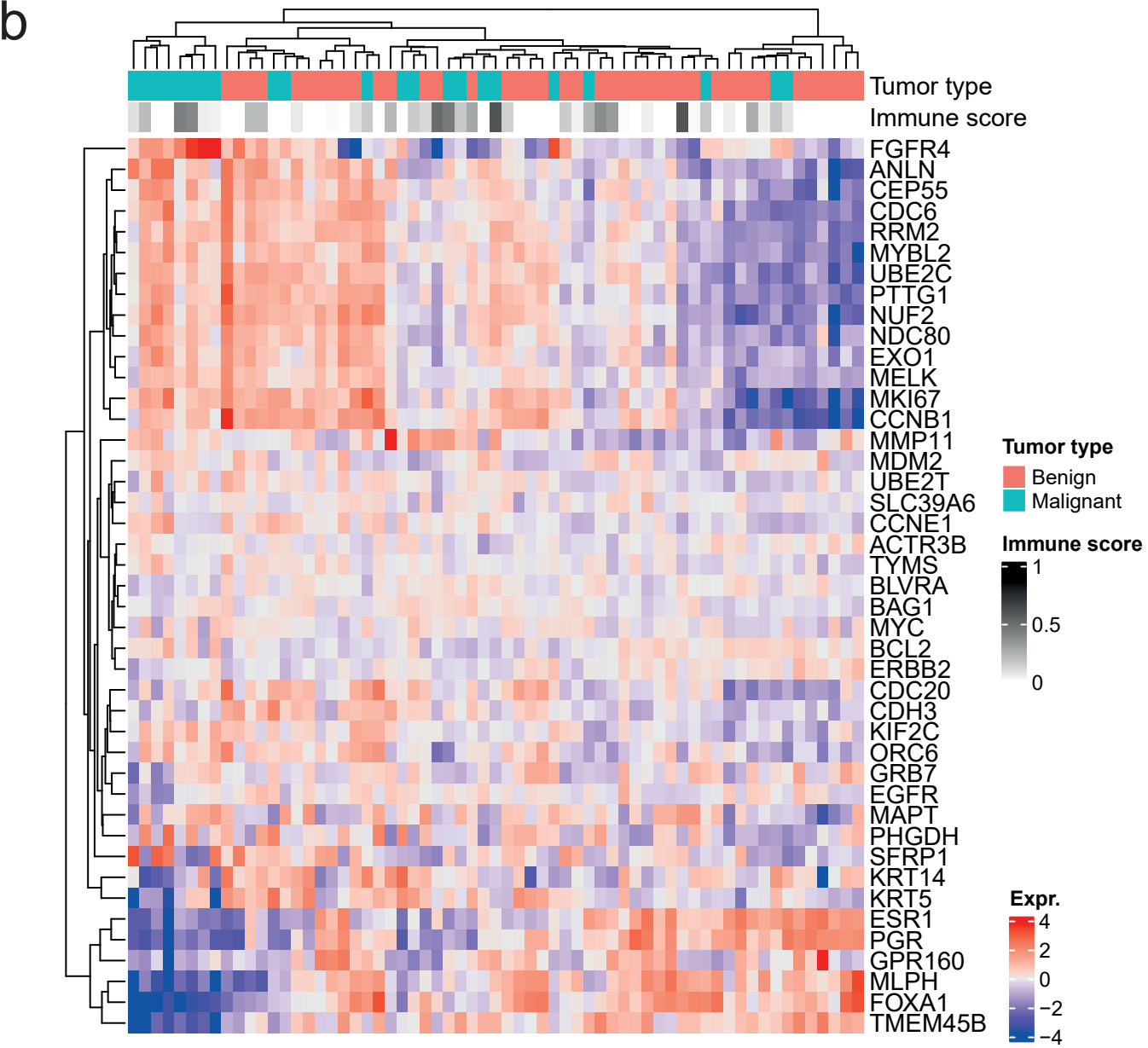

Supplement: Supplementary file 5 — Fig. S5 Hierarchical clustering across the PAM50 genes in two independent canine datasets a: 39 of the PAM50 genes were available in the validation dataset GSE20718 (Klopfleisch et al.) based on Affymetrix gene expression arrays. Tumor samples are shown in columns, genes in rows. Top annotation depicts histological grade, lymph node invasion and immune score b: 43 of the PAM50 genes were available in the validation dataset GSE 136197 (Graim et al.) based on RNA sequencing. Tumor samples are shown in columns, genes in rows. Top annotation depicts tumor type and immune score. (PDF 379 KB) [file 10911_2022_9523_MOESM5_ESM.pdf]

FIGURE S6

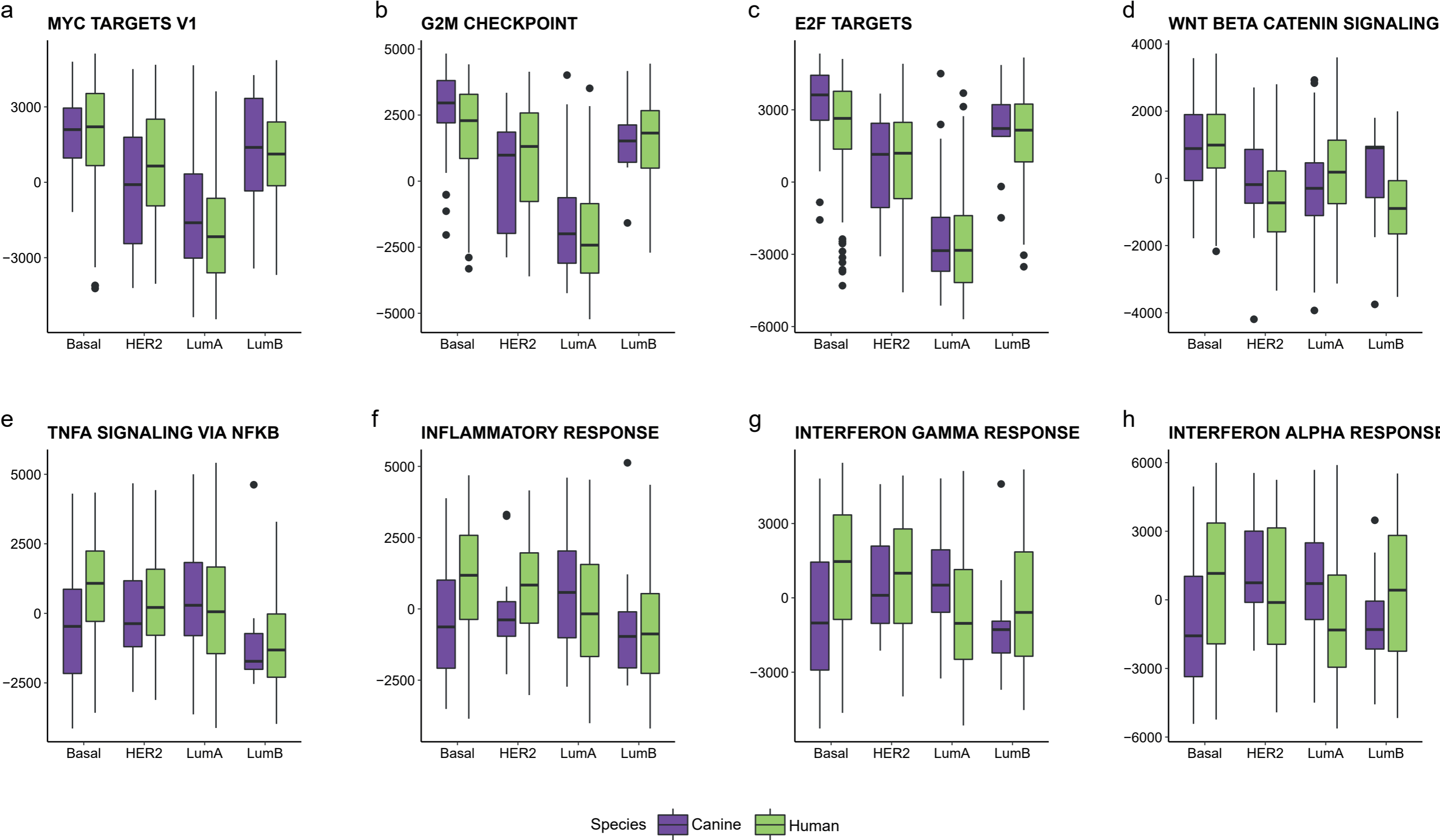

Supplement: Supplementary file 6 — Fig. S6 Expression of Hallmark gene signatures by PAM50 subtype a–d: Hallmark gene signatures that differed significantly between basal-like and luminal A subtypes in both species (Mann Whitney U test, p < 0.001). e–h: Hallmark gene signatures that differed significantly between canine and human basal-like tumors (Mann Whitney U tests, p < 0.001). Single sample gene set enrichment results for all signatures are presented in Supplementary File 4. Boxplots illustrate the median (middle line) and interquartile range (box); whiskers indicate 1.5 × IQR above and below the box. (PDF 198 KB) [file 10911_2022_9523_MOESM6_ESM.pdf]

FIGURE S7

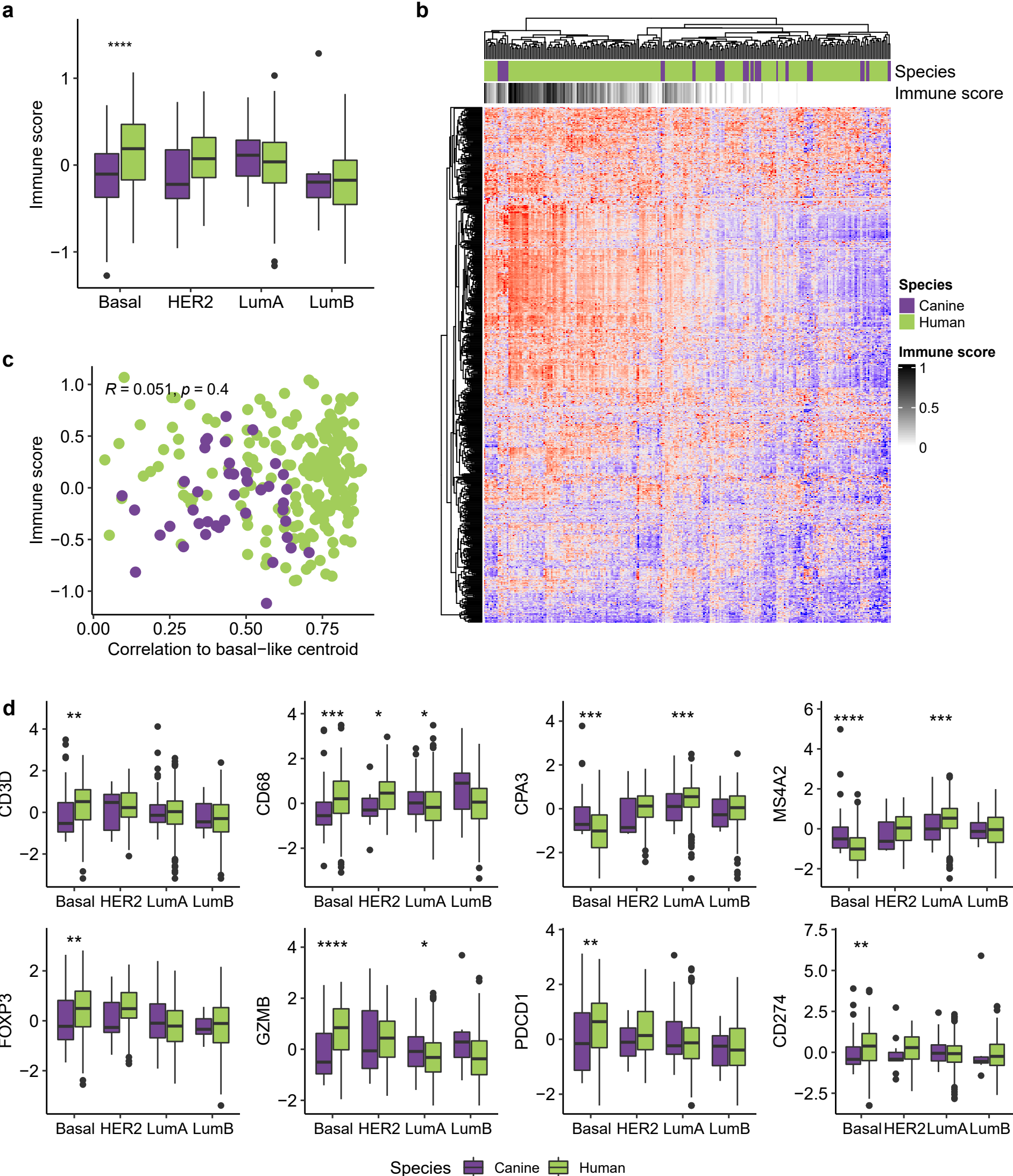

Supplement: Supplementary file 7 — Fig. S7 Immune characterization of CMGT and TCGA a: Immune score by PAM50 subtype in CMGT and TCGA. b: Cluster heatmap showing expression of 650 immune genes in basal-like CMGT and TCGA tumors. Tumors are shown in columns and genes are shown in rows. Clustering of genes was performed using Euclidean as distance metric and complete as clustering method. Top annotation depicts species and immune score. c: Immune score vs. correlation to basal-like centroid in basal-like CMGT and TCGA tumors. Color indicates species. Correlation coefficient and p-value obtained by Pearson correlation. d: Expression of genes characteristic for immune cells in CMGT and TCGA by PAM50 subtype. P-values obtained from Wilcoxon tests comparing expression in canine and human tumors of same subtype are indicated above (* < .05, ** < 0.01, *** < 0.001, **** < 0.0001). Boxplots illustrate the median (middle line) and interquartile range (box); whiskers indicate 1.5 × IQR above and below the box. (PDF 7.48 MB) [file 10911_2022_9523_MOESM7_ESM.pdf]

# FIGURE S8

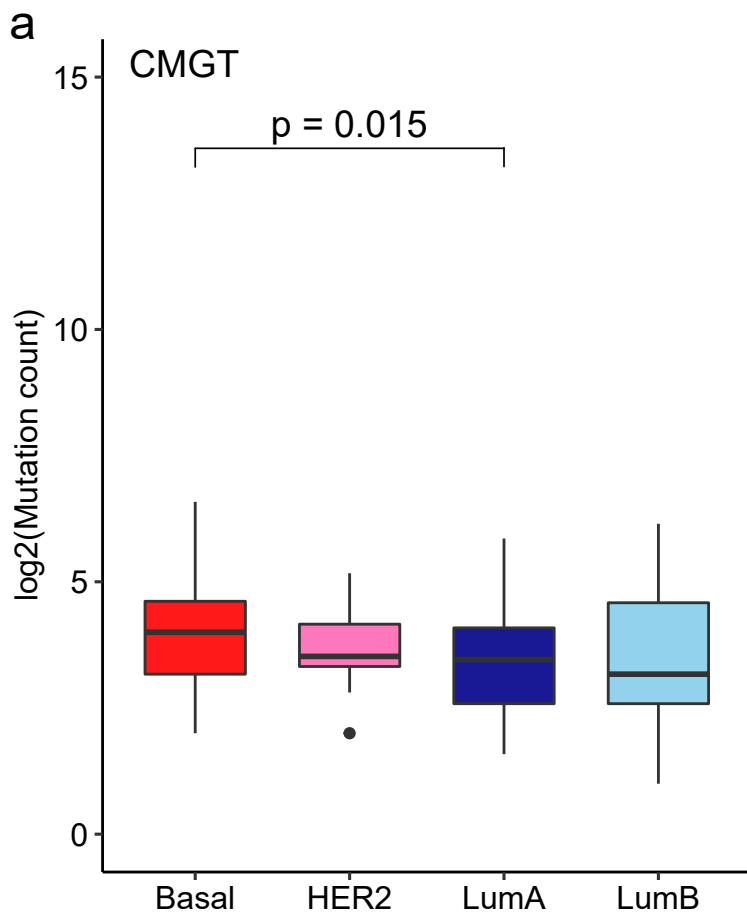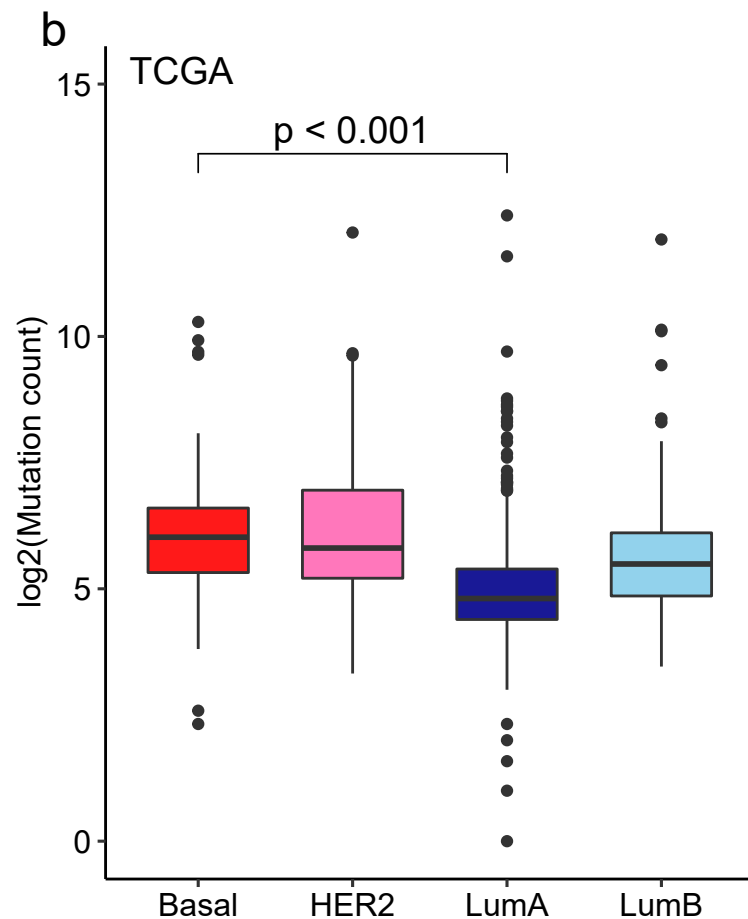

Supplement: Supplementary file 8 — Fig. S8 Tumor mutation burden in a: CMGT and b: TCGA tumors by PAM50 subtype Subtype is shown on the x-axis and log2 of mutation count (number of coding mutations) is shown on the y-axis. P-values are obtained from Mann Whitney U tests comparing tumors of basal-like and luminal A subtypes. Boxplots illustrate the median (middle line) and interquartile range (box); whiskers indicate 1.5 × IQR above and below the box. (PDF 119 KB) [file 10911_2022_9523_MOESM8_ESM.pdf]

FIGURE S9

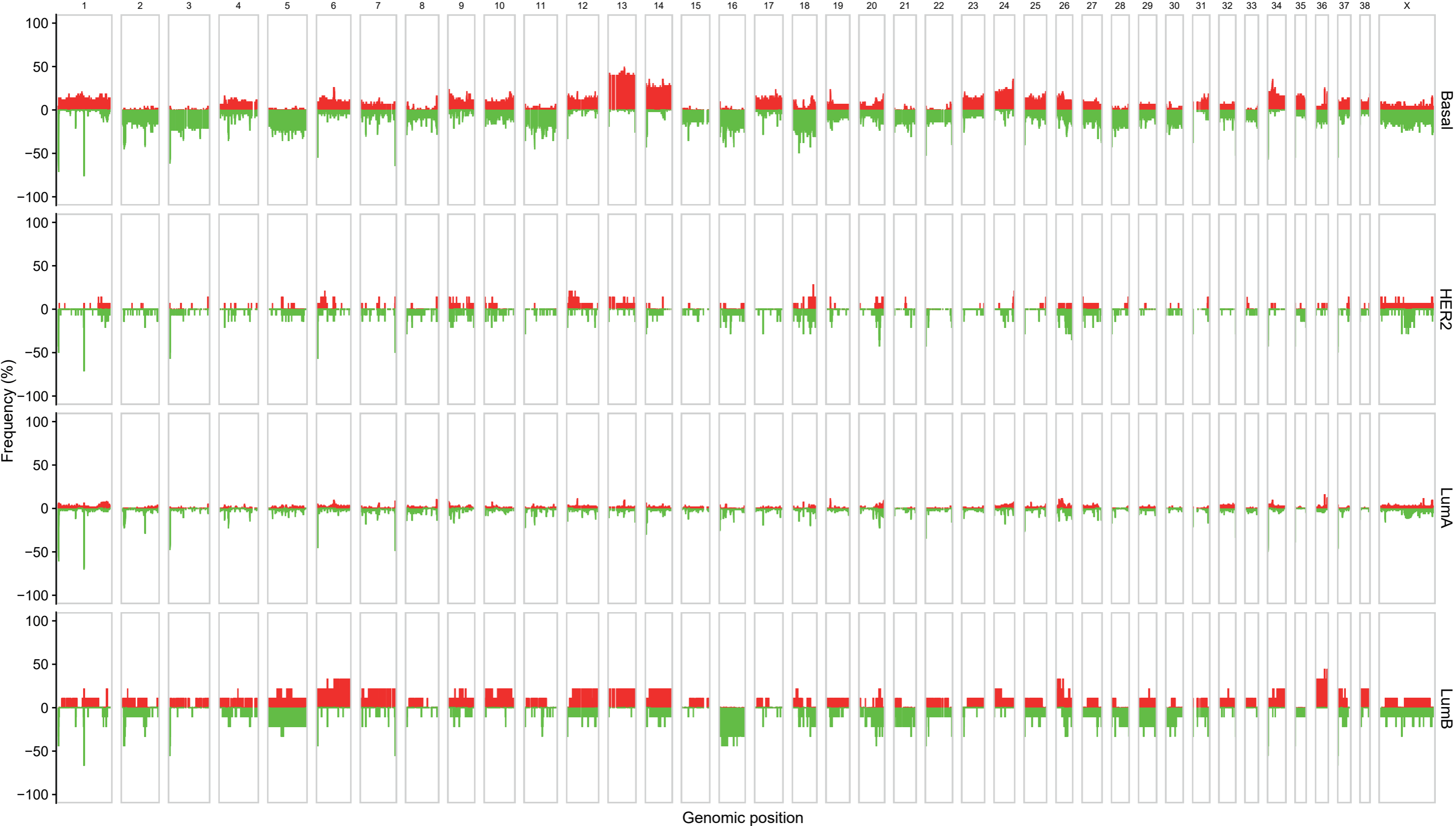

Supplement: Supplementary file 9 — Fig. S9 Genome-wide copy number frequencies by PAM50 subtype in CMGT Genomic position is shown on the x-axis. The y-axis shows the frequency of losses (green) or amplifications (red) in the four subtypes separately. Cut-off for amplification was set at segment mean > 0.2 and for deletion <-0.2. (PDF 1.90 MB) [file 10911_2022_9523_MOESM9_ESM.pdf]

FIGURE S10

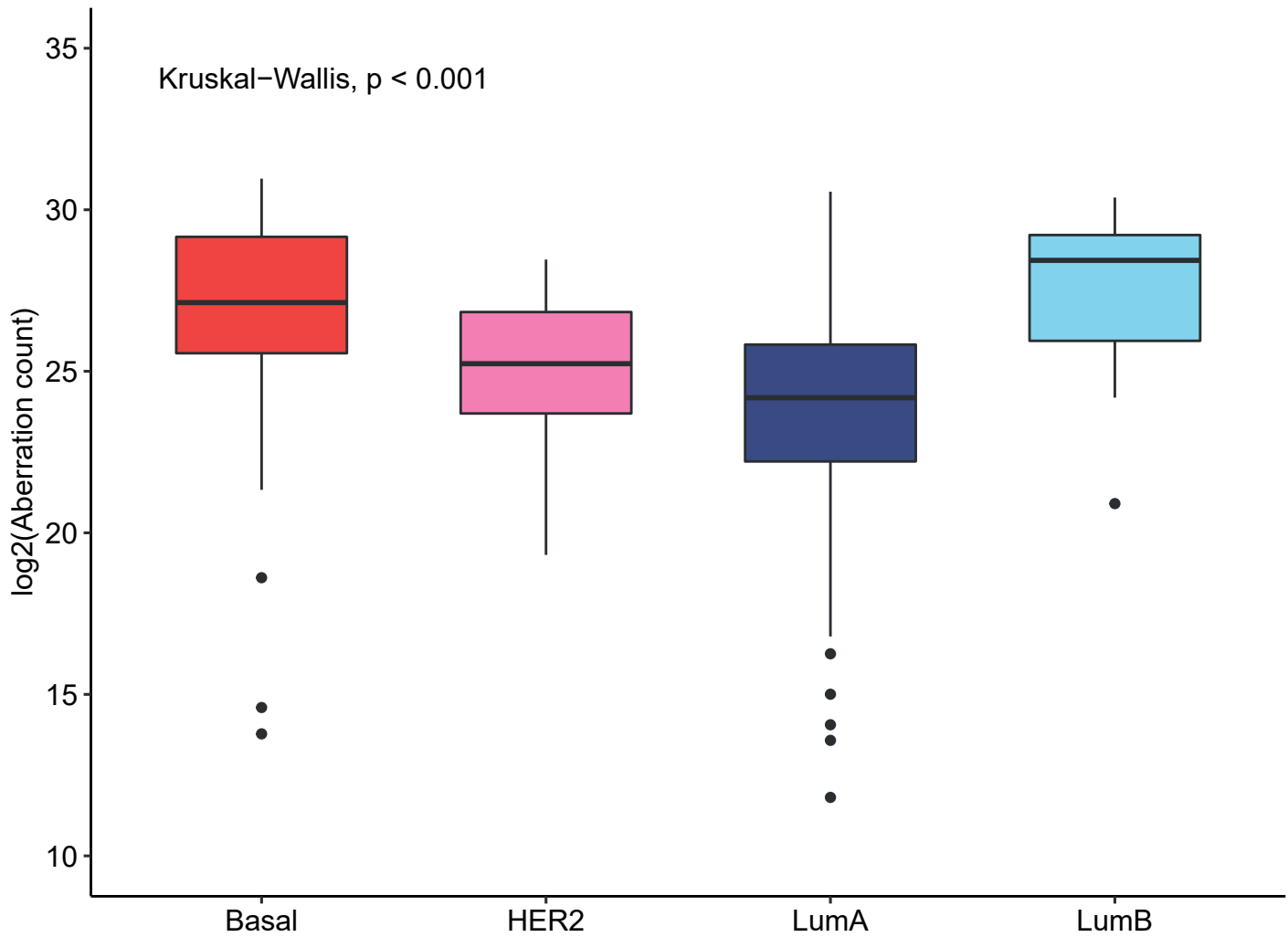

Supplement: Supplementary file 10 — Fig. S10 Copy number aberration count by PAM50 subtype in CMGT Subtype on the x-axis and log2 of the number of aberrations on the y-axis. Boxplots illustrate the median (middle line) and interquartile range (box); whiskers indicate 1.5 × IQR above and below the box. (PDF 196 KB) [file 10911_2022_9523_MOESM10_ESM.pdf]
